# Perspectives of Community Health Center Employees on Public Bus Transportation in Rural Hawai‘i County

**DOI:** 10.3390/ijerph23010078

**Published:** 2026-01-06

**Authors:** L. Brooke Keliikoa, Claudia Hartz, Ansley Pontalti, Ke’ōpūlaulani Reelitz, Heidi Hansen Smith, Kiana Otsuka, Lance K. Ching, Meghan D. McGurk

**Affiliations:** 1 Department of Public Health Sciences, University of Hawai‘i at Mānoa, 1960 East-West Road, Biomed D210, Honolulu, HI 96822, USAmcgurkm@hawaii.edu (M.D.M.); 2 Hawai‘i Island Community Health Center, 75-5751 Kuakini Highway, Kailua-Kona, HI 96740, USA; 3 Papa Ola Lokahi, 677 Ala Moana Blvd, Suite 720, Honolulu, HI 96813, USA; 4 Chronic Disease Prevention & Health Promotion Division, Hawai‘i State Department of Health, 1250 Punchbowl Street, Room 422, Honolulu, HI 96813, USA; 5 Oahu Metropolitan Planning Organization, 707 Richards Street, Suite 200, Honolulu, HI 96813, USA; kiana.otsuka@oahumpo.org

**Keywords:** transit, commute, rural, healthcare access, social determinants of health, qualitative research

## Abstract

**Highlights:**

**Public health relevance—How does this work relate to a public health issue?**

- Transportation is a social determinant of health.
- Rural communities are underserved by public transportation.

**Public health significance—Why is this work of significance to public health?**

- This study examines employee perspectives on accessing a rural community health center by bus.
- Employees and patients faced multiple barriers to using the bus to access the community health center.

**Public health implications—What are the key implications or messages for practitioners, policy makers and/or researchers in public health?**

- More reliable and accessible bus service would benefit both community health center employees and patients.
- The community health center can play a role in advocating for improvements to the public bus system to increase access to healthcare, employment, and other critical services and resources.

**Abstract:**

People living in rural communities are typically underserved by public transportation services and face challenges in accessing healthcare, jobs, stores, and other destinations. Understanding the lived experiences of people who use public transportation in rural communities can help to inform a more equitable transportation system. This qualitative study gathered the perspectives of community health center employees about the public bus system for Hawai‘i Island, a rural county in the United States. Using a community-engaged research approach, the evaluation team interviewed 10 employees through either in-person small group interviews or online individual interviews between April and July 2023. Transcripts were coded and analyzed using a thematic analysis approach. While all study participants were selected for their interest in commuting to work by bus, most believed the bus was not a reliable or convenient option. Participants shared their experiences about not being able to rely on the bus schedule, feeling unsafe while walking to bus stops or waiting for the bus, and other barriers to using the bus system. Participants also shared their insights about how a reliable bus system would benefit community health center patients who needed transportation to more than just their medical appointments, but also to places like pharmacies, laboratory services, and grocery stores. These findings can be used to initiate discussions around the ways that community health centers can further address transportation as a social determinant of health and inform transportation providers about how to best plan and invest in transportation infrastructure and services to meet the needs of rural populations.

## 1. Introduction

Transportation is a social determinant of health, impacting the ability to access jobs, education, healthcare, nutritious food, and other critical goods and services. The lack of safe, affordable, and reliable transportation options disproportionately burdens certain groups, including racial/ethnic minority, lower-income, elderly, and rural populations [1]. This contributes to health inequities through multiple pathways. For example, patients who do not have transportation to healthcare services experience negative health outcomes resulting from delayed or missed healthcare [1]. A lack of transportation may also contribute to social isolation, which is in turn linked to poor mental health [2].

In the U.S., public bus systems are disproportionately used by low-income and racial/ethnic minority populations who are more likely to be dependent on bus service [3]. However, one study found that transit agencies often fail to prioritize the needs of bus riders from low-income and racial/ethnic minority populations, instead focusing more broadly on improving the mobility of all residents [3]. More research is needed to understand how to shift resources towards people who depend on bus service and create more equitable public bus systems. By understanding the different mobility needs of bus riders, transit agencies can prioritize bus service improvements [4].

Public bus transportation further needs to be studied in rural geographic contexts because of unique issues and limitations [5]. Residents of rural communities are more reliant on personal vehicles because of limited public transportation services [6]. However, about 6% of rural U.S. households do not have access to a personal vehicle, representing over 1 million households [7]. In addition, residents of rural communities may be unable to drive due to older age, disabilities, and/or the high cost of vehicle ownership [7]. Longer travel distances, increased transportation costs, and time barriers further contribute to the transportation disadvantage experienced by rural populations [8]. The lack of public transportation in rural communities has a greater impact on the mobility of certain groups, including children, older adults, low-income families, and people with disabilities [4].

As rural populations grow, the demand for public transportation increases [9]. More information about the needs and experiences of rural residents is required to inform public transportation service planning and investments [4]. Perceived barriers are often understudied in considering transportation accessibility [8].

Rural communities are medically underserved, and the lack of access to healthcare services contributes to health disparities in rural populations [10]. Community health centers are an important healthcare provider in rural areas, especially for racial and ethnic minority, low-income, and uninsured populations [11]. At the same time, rural community health centers grapple with workforce shortages and face unique challenges in recruiting and retaining clinical staff, such as a lack of community services and amenities [12]. Further research is needed to understand how commuting and transportation options factor in the ability to recruit and retain clinical staff in rural community health centers, given that work commute experiences are linked to overall quality of life and well-being [13]. Improved public transportation options would benefit both employees and patients in getting to and from rural community health centers.

The purpose of this study was to better understand the lived experiences of employees of a rural community health center related to the public bus system. Gathering the lived experiences of people who engage with the public bus system can provide valuable insights into the local context and ways to make the transportation system more equitable [14].

### 1.1. Hawai‘i Transportation Equity Hui

This formative research study was conducted to support the development of the Hawai‘i Transportation Equity Hui, a statewide working group formed in 2021 to address issues around transportation equity by empowering community voices that are often missing from transportation decision-making. Through funding from the Hawai‘i State Department of Health, the Transportation Equity Hui was coordinated by Papa Ola Lōkahi, a non-profit community organization focused on the health and well-being of Native Hawaiians. For the initial stage, a Steering Committee was convened to create a collaborative vision and assess the state of transportation equity in Hawai‘i. The Steering Committee decided that active and public transportation modes were of high interest and sought to better understand lived experiences and elevate marginalized voices. Based on these priorities from the Steering Committee, we engaged in a participatory research approach to develop the study purpose and conduct the study with a community partner, the Hawai‘i Island Community Health Center (HICHC). We further describe the study setting and community-engaged research process below.

### 1.2. Study Setting

Hawai‘i Island is the largest island in the U.S. state of Hawai‘i, spanning over 4000 square miles. While it accounts for 63% of the state’s landmass, only 14% of the state’s population resides there. The entire island comprises Hawai‘i County, with an estimated population of 209,790 in 2024 [15]. Virtually all (98.7%) of Hawai‘i County’s land area is classified as rural, and the majority (59.5%) of the population lives in rural areas of Hawai‘i County [16]. Approximately 4.7% of households do not have access to a vehicle [17].

The Hawai‘i County Mass Transit Agency operates the public bus system on Hawai‘i Island, known as Hele-On. Hele-On contracts companies to provide fixed and flex bus routes, along with other services like paratransit and vanpool [18]. According to the agency, approximately 588,000 passenger trips per year are provided by a fleet of more than 30 buses, vans, and a trolley [18]. Between 2022 and 2025, the bus has been free to ride, with services subsidized by federal COVID-pandemic relief funds. Maps of the 24 fixed and flex bus routes are available on the Hele-On website. There are seven transit hub connections across the island, with nine local routes connecting towns, twelve neighborhood routes to connect residential areas to towns, one cross-island route, one flex route, and one express route that only provides service during the morning and afternoon [19]. Most routes are along major highways and schedule frequency varies widely depending on the route. For example, the cross-island route runs five times per day while neighborhood routes are scheduled to come once per hour.

The HICHC is a federally qualified health center that serves residents at multiple clinic locations across Hawai‘i Island. All patients are provided with medical, dental, and/or behavioral health services regardless of their ability to pay. Of the 36,357 patients served in 2022, 89.5% were living at or below 200% of the Federal Poverty Level [20]. The majority of patients (66.7%) identified as racial/ethnic minority groups, including 22.0% Native Hawaiian/Pacific Islander, 10.6% Asian, 14.1% Hispanic/Latino, and 28.9% reporting more than one race [20].

## 2. Materials and Methods

Through a community-engaged research approach [21], we designed and implemented this formative, qualitative research study with support from the Transportation Equity Hui and in partnership with HICHC. At the time of the study, the lead community partner (CH) was employed as a Community Health Worker in the HICHC Department of Health Equity. The Transportation Equity Hui’s Steering Committee provided input into the dimensions through which they wanted to better understand transportation equity issues. Through meeting discussions and a short online survey, the Steering Committee prioritized learning more about transportation equity issues facing rural communities, workers who had shifts during non-traditional business hours, users of public transportation, and access to healthcare.

Based on these priorities, we formed an Evaluation Action Group to conceptualize, plan and conduct the research study. The Evaluation Action Group consisted of the university-based evaluation team, the Transportation Equity Hui project coordinator, several interested members from the Steering Committee, staff from the Hawai‘i State Department of Health, and the lead community partner from HICHC. Given the multiple areas of interest, we refined the research question to focus on understanding the lived experiences of HICHC employees who commuted to work by public bus, thus addressing the Steering Committee priorities around rural communities, public transportation, and access to healthcare.

A qualitative study design was selected to fit the focus on understanding lived experiences. Evaluation Action Group members recommended offering options for both individual and small group data collection, as well as online and in-person formats, to accommodate employee preferences and work schedules. The action group also provided input on the general interview questions. The lead community partner recommended expanding the study focus to include employees who were interested in commuting by bus due to a limited pool of employees who were currently commuting to work by bus.

The University of Hawai‘i Human Studies Program approved this study (Protocol 2022-00911).

### 2.1. Data Collection

This qualitative study utilized a semi-structured interview format to gather the experiences and perspectives of HICHC employees. A purposive sample of HICHC employees was selected based on: (1) currently commuting to work by public bus; or (2) indicating a desire to commute to work by bus. A study recruitment email was sent to all HICHC employees to introduce the study and request that any interested employees complete a brief survey with their contact information and preferred method of participating (14 responses received). All employees who indicated their interest in participating (*n* = 8) were contacted by the evaluation team, and 6 participants were recruited this way (2 potential participants did not respond to several email requests). To increase the sample size, the lead community partner personally recruited employees through her knowledge of employees who had experience with the bus system. An additional 4 employees were recruited through her personal network.

Between April and July 2023, the evaluation team conducted individual interviews and small group interviews based on the preference of participants. The semi-structured interviews were tailored to whether or not the participant currently commuted to work by bus (Appendix A). A brief survey to gather basic demographic information about participants was distributed by paper or an online survey link based on the mode of data collection. Four individual interviews were conducted via online videoconferencing; two small group interviews were conducted in-person at two locations on Hawai‘i Island (*n* = 2, *n* = 3), and one individual interview was conducted in person. On average, individual interviews lasted 49 min and small group interviews lasted 85 min. For the in-person small group interviews, food was provided and the lead community partner was present to help two evaluation team members with data collection. Online interviews were conducted by two members of the evaluation team. All participants provided their informed consent and received a reusable, insulated drink tumbler (approximate value of $29).

### 2.2. Data Analysis

The interview data were analyzed following the reflexive thematic analysis process described by Braun & Clarke [22]. A thematic analysis approach was suited to the focus on the lived experiences of the interview participants and identifying themes across the interview participants [23].

The interviews were recorded, then transcribed verbatim. Transcripts were cleaned for accuracy and participant names were removed. In the data familiarization phase, two evaluation team members (LBK, AH) read through the full transcripts and wrote memos about initial coding ideas.

In the coding phase, the two team members discussed their coding ideas and developed initial coding categories. Data from the transcripts were then organized in a matrix [24] by summarizing data excerpts from each transcript for each participant. The excerpts were organized into the initial coding categories of current commute to work, reasons to commute by bus, barriers to commuting by bus, personal experiences with the bus system, friend/family experiences with the bus system, patient transportation needs/experiences, and recommendations.

After reviewing the coding matrix, the evaluation team generated initial themes related to participants’ experiences, thoughts, and perceptions of the Hawai‘i Island bus system. The evaluation team presented these initial themes back to participants for feedback through an online meeting as part of a member checking process. Participants who were unable to attend the meeting were sent a summary of the themes and asked to provide any feedback by email. In addition, preliminary findings were shared with the Director of the HICHC Health Equity Department and next steps for the health center were discussed. All feedback was documented and used to refine the themes.

As part of the iterative coding process, one team member (LBK) systematically coded the transcripts using NVivo (Version 14, Lumivero, Denver, CO, USA). The initial coding categories were slightly refined, then the coded extracts were reviewed and used to revise the themes. Co-authors provided additional input into the themes, and this paper represents the final phase in the thematic analysis of writing up the findings.

## 3. Results

### 3.1. Participant Characteristics

The 10 employees who participated in study interviews were all full-time employees at HICHC. They held a variety of roles in the community health center, including clinical provider, behavioral health case manager, community health educator, community health worker, patient navigator, supervisor, and eligibility lead specialist. The majority of participants (*n* = 7) reported currently driving to work, while two walked to work and one commuted by bus. Nearly all participants reported that their current commute to work was under 20 miles (*n* = 9) and took 30 min or less (*n* = 8).

Participants were from diverse gender, age group, and racial/ethnic backgrounds. Half the participants identified as female, while four identified as male and one identified as non-binary. Participants were aged between 18–69 years old, with the most commonly occurring age group being 30–39 years (*n* = 4). Similar to the racial/ethnic composition of the HICHC patient population, about two-thirds of participants (*n* = 7) identified as belonging to non-white racial/ethnic groups, including two who identified as belonging to more than one racial/ethnic group. Half the participants identified as Native Hawaiian or Pacific Islander and one-third identified as Hispanic/Latino.

### 3.2. Qualitative Themes

**Figure 1:**
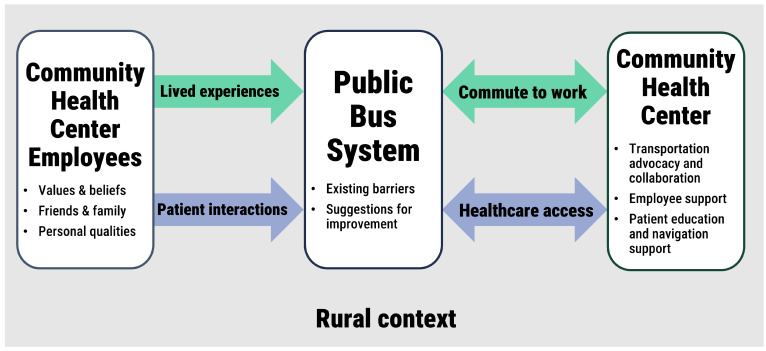
Perspectives of community health center employees on the public bus system related to commuting to work and patient access to healthcare in a rural community.

Interview participants shared a range of perspectives on the public bus system for Hawai‘i Island. Figure 1 shows how these perspectives were organized along two pathways relating study participants, the public bus system, and the community health center. The interrelated components of the diagram are italicized in the following description. Participant perspectives were influenced by their values and beliefs, what they heard from friends and family members, and personal qualities such as where they lived and their gender. Their *lived experiences* with the bus system shaped their perspectives on *commuting* to health center locations by bus. Participants also shared insights shared from their *interactions with HICHC patients* about how the bus system impacted patient *access to healthcare*. While the first pathway aligns with the primary research question, the second pathway emerged from what participants felt compelled to share about the needs of patients. Common barriers to using the public bus system were discussed, and participants recommended ways to improve the existing bus system. The last section of the diagram reflects a bidirectional relationship between the bus system and community health center: participants proposed ways that the community health center could better enable employees to commute by bus and help patients access healthcare and other resources. The *rural context* was a consideration for all aspects of the diagram. Themes related to this conceptual study diagram are discussed in further detail below.

#### 3.2.1. The Public Bus System Is a Valued Community Resource

Overall, participants had favorable views on bus transportation and shared that they liked using public bus systems in other places, such as Honolulu, Seattle, and New Zealand. Participants shared multiple reasons for why they wanted to be able to commute to work by bus. The primary reason was saving money because of the high costs of fuel and owning a vehicle (e.g., insurance, maintenance). Participants also described not wanting to experience the stress of driving in traffic or trying to find parking, which was limited or off-site at clinic locations. A reliable bus option would also help families who were juggling work and school schedules and trying to coordinate drop-offs and pick-ups. One participant described the coordination needed around sharing the one family vehicle:

*“We have to make schedule, we have to arrange, because you only have one transportation. We have to either leave the car, or my wife drop me off, or yeah, she usually drop me off if we have appointment later on in the afternoon, in a day so that she can come to her appointment, or our kids’ appointments… but if we, I would have the bus, I would’ve just take the bus, come to work because then she will use the car for health appointment, or other stuff.”*

(P5)

Most participants had some experience with the Hele-On bus system on Hawai‘i Island. Positive experiences with riding the bus typically involved leisure trips, such as going to a shopping mall to watch a movie and riding to another side of the island to hang out with family members on the weekend. One participant described catching the bus as convenient to avoid the hassle of trying to find parking in a busy lot at a big-box retailer; however, the route to return home by bus would have taken too much time, so they used a rideshare service. The overall takeaway from the experience was positive:

*“I just rode it from where I live to town and then get an Uber back home, you know. That’s what I did then. I just did it just for the fun of it, just to see what the experience is. Now, you know, riding the bus it’s—I think it’s just a good thing for us, and I’m grateful that we have bus transportation that it covers a lot of the areas nowadays.”*

(P3)

Participants who regularly used the Hele-On bus system were appreciative of the bus service and felt that the drivers had a lot to handle with difficult passengers, traffic, and other challenges. One participant said that he observed the bus drivers having to deal with passengers who were drunk, illegally vaping, or listening to their electronic devices without earphones:

*“Honestly, I told them that I really respect them after, because when I used to [drive] behind the bus, I was like, ‘Come on, man, pull over. I got to go.’ But when I see what they’re going through on a daily road, I was like, ‘I feel sorry for these guys. I really appreciate them.’”*

(P10)

#### 3.2.2. Commuting to Work by Bus Is Not a Feasible Option

While all participants were invited to participate in this study because of their interest in commuting to work by bus, most participants thought that it was not currently feasible to do so. One of the main concerns was around the perceived lack of reliability of the bus arriving on schedule. For example, when a participant decided that she wanted to try catching the bus to work, she had to give up so that she would not be late to work:

*“About six years ago, there were gas prices that were just went crazy and in protest, I was going to catch the bus to work. I just was like, ‘That’s so unfair.’ I wasn’t going to take it. I had my husband drop me off down at the bottom of our subdivision, so I could wait for the bus, and for whatever reason, either it was late, or it was early, or something, but I waited there probably for about 15 min during the time I was supposed to have…and I was like, ‘I got to go to work.’ I called my husband, he came to pick me up, and then I took my car to work.”*

(P6)

Another participant shared that they heard from multiple people that in addition to the bus not coming on schedule, the bus also did not reliably pick up/drop off passengers in designated locations. One participant relayed the experience of a friend:

*“She’d tell me about, you know, like how early she’d have to leave to get on the bus because the bus could show up 20 min earlier, it could show up 20 min late, or it could not come at all. And then she’d say, there’s times where she would get on the bus, and then the bus driver would drive by people on the side of the road that were waiting for the bus, and just wouldn’t pick them up. And every day that she would get on the bus, the bus would take a different route. So you never knew. Like even it was the same number. You didn’t know if that bus was gonna go through that neighborhood.”*

(P2)

The rural context of Hawai‘i Island presented additional barriers. Several participants mentioned that the bus did not come into their neighborhoods, so they would have to walk out to a highway to get to the bus. Usually, there was no sidewalk or safe space to walk along the road to get to the highway. The lack of official bus stops and trying to flag down the bus to ride was also perceived as a barrier to using the bus.

*“I do not believe there is a bus stop in my neighborhood and I honestly don’t know where any of the bus stops are, like they’re not very well marked and I often see people just standing on the side of the road waving for the bus, and I’m like I don’t know if I would do that—I don’t know. Is that an actual bus stop, I don’t know, like I just feel like there’s so many things about the system that I don’t know, or like just don’t exist because it’s so rural.”*

(P2)

#### 3.2.3. Women Were Concerned for Their Personal Safety

Personal safety was an important consideration for participants who did not identify as male, particularly safely getting to/from the bus pick up location and waiting for the bus along the road or highway—often far away from where people lived. Because bus stops were not always officially designated with a sign and other amenities like a bench, it was described as standing by yourself in a small space on the side of the road. Participants explained that it could feel unsafe because of the other people in the area. One participant described hiding away from the road at night:

*“Back when I was riding [as a teenage girl], but when I would feel sketched out, I would hide. Like I would be on the top of the highway or something at night, and I would hide in the bush, or like somewhere where I can see out but nobody can see me, just to avoid any person or anybody that could possibly stop and try to mess with me or something, you know…I didn’t really trust, I didn’t want to chance it.”*

(P4)

One of the male participants acknowledged the role of gender in feeling safe while using the bus system. He usually tried to pick up his girlfriend from the bus stop if it was late, so that she would not have to walk home from the bus stop in the dark. He shared a scary experience that she had one night:

*“She was walking home [from the bus stop] and this spooky looking guy who turns out he’s kind of a transient or a homeless guy who is kind of in the area. Sometimes he sleeps up on the ground at the top of the block. Well, he was standing there in the middle of the road once, so she walked by, and you know for me I wouldn’t care, but for her she got freaked out, and then she thought he started walking towards her, so she ran all the way [home]… And I actually called the police because she was so scared. So you know, I think maybe it was an exaggeration on her part, but you don’t know, you don’t know if someone’s gonna accost you right.”*

(P1)

#### 3.2.4. Patients Need a Bus System That Enables Them to Access Healthcare and Other Resources

Transportation was a significant challenge for many patients of the community health center, so participants wanted a more reliable, safe, and accessible bus system that met the needs of their patients. Participants mentioned that many patients of the community health center lacked access to a personal vehicle and relied on the bus or friends and family members to get to medical appointments.

*“Because we’re a Federally Qualified Health Center and because we serve people regardless of their ability to pay, many of them are going to be without vehicles. And if they had reliable transportation like a bus that they could get on for a nominal fee, or a monthly fee like they have on O‘ahu, or even free like it is right now, it would make it easier, I believe, for them to get on the bus and come to see us. And we would be able to rely on their meeting their appointments and getting to their appointments on time if they had something that was reliable like transportation because that’s our biggest issue on this island is transportation. And so it’s hard—it’s so expensive to own a vehicle because you’re not only purchasing it, you’re maintaining it, you’re putting fuel in it, you’re paying insurance on it, you’re doing safety checks on it… registration on it and registration has gotten crazy… you’re putting tires on it and it’s like, oh my gosh, it’s just so expensive. Who can afford that if you’re…not even making a living wage. Nobody’s really making living wages here. So, you know, the people that our clinics are the safety net for would hugely benefit from having the opportunity to catch public transportation that was reliable and convenient and went in [to the neighborhoods where people live].”*

(P6)

Patients who did not have family members, friends, or neighbors to help with transportation sometimes found themselves stranded because of limited bus service. One participant described having to help a patient, but in an unofficial capacity:

*“…One day we had a patient telling myself, and this was really late, it was probably eight, nine at night. And he was discharged from the ER, yeah, the ER, but he had a broken arm so he couldn’t drive. So, no bus so he called me and he called [co-worker] just to, and we were like, because I have my daughters. And I was like, I cannot go right now. And she said like, ‘Oh, I can go.’ So, we’re pretty much the bus here or the taxi service sometimes. But that was, and the other thing just because under the health center policy, we are not allowed to transport patients. So that was at night. So, we were like, okay, like we’re not on the clock right now. We can do this, but otherwise he will have to walk.”*

(P9)

Patients who do ride the bus to the community health center might miss their appointments if the bus comes late, early, or not according to schedule because of the large gap between scheduled bus services. For medical appointments with fixed time slots, relying on the bus for transportation might result in delayed care.

*“…Because on our schedules we only have certain time slots, so when we offer a time slot, the person will, or the patient will say, ‘No, I gotta catch the bus—bus doesn’t come till this time.’ And there’s times where either we have to give them a option either, you know, like either we look for another date, another time. So postponing right? … If we are seeing a patient that relies on public transportation with no other method of transportation available to them, then that results in either rescheduling the appointment, postponing it or you know, it just delays access to their health services pretty much. That’s what happens and then, of course, we have walk in and same day appointments, too, but that can fill up by like 7 in the morning. So we have to tell them like, ‘Well, we have walk in and same day, but gotta be here by 7.’ So we’re still getting them a time constraint. And you know, so it definitely postpones patients’ health services. That’s for, like our set provider schedules for medical, behavioral health, dental… I know it can be difficult for patients who rely on public transportation as their primary method.”*

(P4)

Although participants did not have confidence in the bus system being a reliable mode of transportation for their patients, they did print bus schedules and share information to help them navigate the bus system. One participant also mentioned that they would try to give them insight into where they were going, such as landmarks or if they needed to be cautious about anything. When sharing information about the bus, participants felt that it was important to share that the bus might not come on time and to manage their expectations.

*“I guess, based on my experience I’m biased, and… I would warn people like, ‘Hey, it’s possible that you know, like the bus might not come on time.’ You just kind of let them know like if you’re gonna catch the bus, try to get there extra early and make sure you hang out longer than you think and you know I just—I try to manage people’s expectations. Am I going to tell somebody to not try it, if that’s their only mode of transportation? No, I’m going to be like, ‘Please try the bus, like you need to figure out how to use the bus so that you can have your independence essentially.’ You know what I mean? Like for people that don’t have cars it’s like I want to support them, and in being independent and empowering them to use the resources. So, even though I have a bias like, I would just use that for managing expectations, not necessarily telling people to not use the service, you know.”*

(P2)

Patients further experienced additional challenges with accessing transportation services, including language barriers, lack of service availability, and inability to access existing services. Interview participants described the complex, intertwining nature of the barriers facing their patients. Existing transportation services (e.g., paratransit) were hindered by a lack of drivers and limited geographic coverage, resulting in long wait times or infrequent availability. For patients with mobility limitations, the bus stops were not accessible because of long distances to walk from the patient’s home to the bus stop out on the main highway. A lack of sidewalks further made it more challenging to safely walk to bus stops. If patients had Medicaid insurance, then they could use a medical taxi to get to appointments. However, the medical taxi could only be used for medical appointments; thus, patients did not have transportation to lab services, pharmacies, and grocery stores to cover their other needs. For elderly patients with Medicare insurance, they did not have any transportation benefits, so participants described how much they struggled to help these patients find transportation to medical appointments.

*“This is how bad it is with the transport because there’s no transportation available right? So, she’s an elderly woman and one of the providers was seeing her. This woman continues to have seizures after seizures, after seizures. So Doc came to me and was like, ‘Look, [participant name], I hate to do this, but she can’t drive. And I’ve told her this many times and she continues to show up at the clinic in her car, she drives herself here, and this is, it’s very dangerous. She can hurt someone.’ So then we have to do what we have to do, follow the steps to remove her license and all of that, but people are having to drive, they know that they shouldn’t be driving. My point is she knew and because when I told her, and I approached her and I said, ‘Look, this is the situation.’ She said, ‘Yeah, I do understand, but do you understand that if I don’t drive, what am I going to do? I have things to do. I don’t have people to drive me to the grocery store, to go get my medications, so what am I supposed to do? Just stay home and just wish things magically fall in my hands, like my medications and my food and all my needs.’”*

(P8)

#### 3.2.5. Bus Service Needs to Be More Reliable, Convenient, and Accessible

Based on their personal experiences, as well as their knowledge of patient needs and the experiences of friends and family, participants identified many ways to improve the bus system to be more reliable, convenient, and accessible. Their suggestions were organized into several categories that included improving service coverage, designating official bus stops, and making service more predictable (Table 1).

#### 3.2.6. Supports the Community Health Center Could Provide to Employees and Patients Around Bus Transportation

Participants wanted to advocate for improved bus transportation to support their patients, viewing transportation as an important strategy to address health equity. One participant shared that they recently met with the Mayor to request a bus stop near one of the HICHC clinic locations. However, additional advocacy efforts were warranted, especially to support lower-income patients who did not have health insurance that covered medical transportation and patients living in more remote communities of the island.

*“I’d like to advocate for all of our patients and people that we work with. Transportation is a big issue here on the Big Island. And for me to be able to get a patient from one place like to go to the specialist, to go see an eye doctor, I mean just everything…It’s really hard for them to get anywhere basically here on Big Island. I feel like if you don’t have your own vehicle, you’re pretty much, you’re not able to do a whole lot because of the transportation situation here. We don’t have transportation.”*

(P8)

One of the first steps that participants recommended was initiating discussions around employee commuting and transportation issues with HICHC leadership. Participants suggested presenting a “wish list” of options that would facilitate commuting by bus. For example, one suggestion was coordinating work assignments so that community outreach activities requiring the use of a vehicle would fall on specific, scheduled days; that would enable employees to commute by bus on the days that they were strictly in the clinic setting. Flexibility in start times would accommodate employees who were late because the bus did not arrive on time. As one small group discussed, one benefit to the employer would be not having to pay for parking to accommodate their employees:

*“The current way of the bus, there would be no way this employer would support it. We’d never get here on time… I don’t imagine them promoting the way the bus runs now.”*

(P7)

*“The way it is now, yeah. But if they, we had more routes, more buses reliable, yeah, they definitely would, because, I mean part of the reason, they do have to pay for, since parking is also so limited here in town, they have to pay for parking, for employee parking for us to park there and then walk up to the clinic. So, I’m thinking they probably would like the idea of us catching a bus to work…”*

(P8)

To support HICHC patients, participants suggested that the community health center could collaborate with other community organizations to share information about existing transportation services and available programs. Participants were also interested in receiving training that would better enable them to educate their patients about bus services and developing informational materials that could be given to patients or shared at outreach events. Another idea to help patients navigate the bus system was to have experienced riders share their tips and lessons learned. For example, study participants shared that it was helpful to call the phone number on the Hele-On website to check the status of a bus and described how to wave down the bus so that it would stop to pick you up along the roadway.

## 4. Discussion

The lived experiences of study participants, along with those of their family members, friends, and patients, shaped perceptions about the Hele-On bus system on Hawai‘i Island around reliability, convenience, and safety. Their experiences confirm the overall need to expand public transportation services for rural communities [25]. Barriers to accessing the community health center by bus impacted both employees who needed to get to work and patients who were seeking healthcare services. The insights shared in this study can be used to improve bus service and inform priorities around resource allocation.

Previous research has found that inconvenient schedules and infrequent service were the most common barriers for residents of rural communities to use public transportation for healthcare trips, suggesting that better coordination between transit and healthcare providers would be beneficial [26]. Study participants believed that their community health center could help to advocate for improvements to the bus system, and their insightful knowledge of the transportation challenges facing their patients would be valuable to inform improvements. Having public bus transportation as an option for their patients would take the burden off the health center for having to provide or manage its own transportation program. Sharing these study findings with County leaders would be a first step towards advocating for increased funding and improvements to the Hele-On bus service. Within HICHC, study participants wanted additional training and materials that would better prepare them to assist their patients with using the bus system. One next step would be identifying a champion to lead coordination and outreach efforts around the public bus system for HICHC [6].

In addition to reliability and convenience, lived experiences also shaped perceptions around safety. Personal safety encompassed several dimensions, including a lack of infrastructure that allowed participants to safely walk to bus stops and feeling unsafe while waiting for the bus or walking between the bus stop and their destination. Previous research has found that women have distinct safety and security needs in urban transit environments that require additional consideration by transit providers [27]. This study confirms that those safety and security needs are also important in rural communities where bus stops are in isolated areas. Good lighting and use of security technology were recommended interventions at bus stops.

The current bus system was perceived as too challenging to be a viable commuting option for study participants. For it to be feasible to commute by bus, study participants offered practical suggestions to improve bus service. Many of their suggestions have already been identified in the county’s Transit and Transportation Multi-modal Transportation Master Plan [28]. Implementation of the master plan would support more predictable bus service. Participants also mentioned observing some encouraging improvements, such as seeing bus stop signs being installed to mark official bus stops. However, increasing access to healthcare services was not a specific objective in the master plan. Future updates to the master plan could better integrate healthcare stakeholders and incorporate feedback from this study. For example, a few participants were confused by flex routes and preferred fixed routes with designated stops. A potential next step would be facilitating a meeting between HICHC employees who use the bus, HICHC leaders, patients who rely on the bus, and Hele-On program staff to discuss ways to improve access to HICHC clinic locations through bus transportation.

Additional research around public transportation experiences in rural communities is needed to better understand community needs. This study was conducted in a rural island county and findings may not be generalizable to other rural contexts. The participants in this study were demographically diverse, but their perspectives may not be representative of all HICHC employees because they were purposively recruited for their interest in commuting by bus. Moreover, only one participant regularly commuted to work by bus. This may be indicative of how challenging it is to use the bus to commute to work. Future research with HICHC patients who rely on the bus would further illuminate how the public bus system intersects with their ability to access healthcare services and quality of life.

## 5. Conclusions

Increasing access to public transportation is an important health equity strategy [29]. Residents of rural communities need bus service that is reliable, convenient, and safe, and the lived experiences of residents who rely on the public bus system can inform ways to direct limited resources towards bus service. Community health centers can help to advocate for transportation improvements to further address social determinants of health for their patients.

## Figures and Tables

**Table 1:** Suggestions for improving the Hawai‘i Island bus transportation system.

Barrier	Suggestions for Improvement	Illustrative Quote (Participant ID)
Lack of accessibility to bus service around homes and important destinations	Adjust bus routes to enter subdivisions, rather than stopping just along major highways	*“I come up this place [name redacted] where the buses won’t go up that subdivision and people have to walk down, sometimes miles down to the bottom of [the highway], and it has a place that the bus can pull off for people, but you have moms and babies and strollers on the side of the road with no covered area and they’re waiting for the bus. Sometimes when I’m passing, I’m thinking, ‘I’m so happy it’s not raining,’ because who knows how long the bus takes to go pick them up. But these are families, they’re on the side of the road waiting for buses to come and get them in the elements.”* (P6)
	Create more local service loops with stops near schools, stores, and healthcare	
	Provide safe infrastructure (e.g., sidewalks or walking paths) to improve access to bus routes	
Lack of bus stops and amenities	Add signage to designate official stops	*“But improvements like I said earlier was the bus stops. Like in my subdivision, having bus stops would be a great. It’d be great to sit down for one thing. I see people waiting, and like even elderly, a lot of elderly use the bus, and they’re standing there with all of their bags, and they’re waiting for the bus, you know. So definitely, bus stops in the subdivisions would be amazing, even if the bus stops itself had its own street light, you know. Have a light on top of the bus stop somehow, solar-powered… because it’s in a subdivision there’s not security like a shopping center that has a public bus stop. So having some type of access to emergency services, either utilizing a button, a button that’s linked or attached to the bus stop that that activates emergency services or having an emergency phone that’s just for security to be able to make people feel more safe and not everybody has a phone.”* (P4)
	Add bus shelters to provide shade and rain cover	
	Add benches or seating	
	Improve lighting and add safety features	
Unpredictable service	Provide information about real-time tracking of buses (e.g., create a bus app)	*“I think a little more technology on the bus would definitely help knowing when it’s really going to be there. As a parent, if it’s really going to come today, then you could take it, if it’s really not, then let me do the work [to drop off child at school], but we don’t use it as an option at all because we don’t rely on it.”* (P7)
	Increase service frequency and reduce waiting times between bus services	
	Prioritize fixed routes over flex routes	
Poorly maintained buses	Regularly maintain buses to prevent mechanical breakdowns	*“I’m getting to know more people on the bus, because I actually asked them, ‘Hey, what do you guys like and don’t like about [the bus],’ before we start this [interview], so I would actually ask… The majority of them were saying, ‘Maintenance,’ they don’t like. The bus is not on time because it’s not upkeep.”* (P10)
	Improve ventilation to minimize diesel odors and ensure that air conditioning is functional	
Lack of information about bus services	Improve website so that it is easier to understand and find information	*“I’m also thinking about those low-income families. They only have one transportation or even no transportation. They’re living in the public housings and apartments… If they get more information about the bus schedule and about bus services on island, then they would utilize that services.”* (P5)
	Provide translated informational materials (e.g., Spanish, Marshallese)	
	Conduct outreach with populations who would benefit from bus service (e.g., seasonal agricultural workers, recent immigrants)	

## Data Availability

The data presented in this study are available on request from the corresponding author due to ethical considerations.

## Abbreviations

The following abbreviations are used in this manuscript:

HICHC	Hawai‘i Island Community Health Center

## Appendix A

### Guiding Interview Questions

The individual and small group interview questions were tailored to whether or not the participant currently commuted by bus. General interview questions included:

For participants who currently commute by bus

- Please tell us about your commute to work. What is it like getting from your home to the health center?
- What does your commute typically involve when you get off work?
- Why do you use the bus to get to or from work?
- What do you like about getting to or from work by bus?
- What is the most difficult part about getting to or from work by bus?
- What would make it better for you to get to work by bus?
- Do you have any additional thoughts about improving your work commute by bus?

For participants who would like to commute by bus but currently do not

- Please tell us about your commute to work. What is it like getting from your home to the health center?
- What does your commute typically involve when you get off work?
- Since you do not currently use the bus to get to or from work, why are you interested in commuting by bus?
- What are some of the reasons why you currently do not commute to work by bus?
- What would make it possible for you to get to work by bus? What are the changes that are needed to make it safer, more convenient, and/or more feasible for you to commute by bus?
- Now that you have shared your experiences with us about the bus system, how do you think that applies to clients who need to or want to catch the bus to get to HICHC locations?
- Do you have any additional thoughts about improving the Hele-On bus system?
